# Reclaiming streets for outdoor play: A process and impact evaluation of “Juega en tu Barrio” (Play in your Neighborhood), an intervention to increase physical activity and opportunities for play

**DOI:** 10.1371/journal.pone.0180172

**Published:** 2017-07-03

**Authors:** Andrea Cortinez-O’Ryan, Andrea Albagli, Kabir P. Sadarangani, Nicolas Aguilar-Farias

**Affiliations:** 1 Department of Physical Education, Sports and Recreation, Universidad de La Frontera, Temuco, Chile; 2 Division of Health Planning, Ministry of Health, Chile; 3 School of Kinesiology, Faculty of Health Sciences, Universidad San Sebastian, Santiago, Chile; 4 Department of Physical Education, Sport and Human Movement, Autonomous University of Madrid, Madrid, Spain; Pennington Biomedical Research Center, UNITED STATES

## Abstract

**Background:**

New strategies are required to create supportive physical and social environments for children and promote active free-play. Juega en tu Barrio (JETB; Play in your Neighborhood) was designed and implemented to explore the effectiveness of closing a street in a low-to-middle income neighborhood in order to increase children’s outside play and physical activity.

**Methods:**

A pretest-posttest design with control group was employed to investigate the intervention effects in a subsample of 100 children, 51 from the intervention neighborhood and 49 from the control neighborhood. The children wore pedometers for one week, and their parents completed questionnaires at two time points: before the intervention began and during the last two weeks of the intervention. JETB was conducted in the intervention neighborhood from 17:30 to 20:30, twice a week, from September to December 2014. Stewards ensured that the children were safe. Children and adults were assessed using systematic observation.

**Results:**

The intervention and control neighborhoods included 177 and 116 children respectively. The average attendance per event was 60 children (SD = 22, reach 34%). In the intervention neighborhood, a significant increase between baseline and final assessment was observed in after-school outdoor playtime (p = 0.02), steps during the 3-hour intervention (p = 0.004), and daily steps Monday to Sunday (p = 0.006). Meanwhile, no changes were observed in the control neighborhood for the same variables. The proportion of children who met recommended daily step counts increased from 27.5% to 53.0% in the intervention neighborhood (p = 0.007), while for control neighborhood no difference was observed (49.0% to 53.0% p = 0.804).

**Conclusions:**

JETB showed high community engagement while offering opportunities for increased outdoor play in children. The intervention showed a significant effect on the number of children meeting the daily pedometer-derived physical activity recommendations.

## Introduction

There is strong evidence for the beneficial effects of physical activity (PA) on children and young people’s health outcomes such as adiposity, cardiorespiratory fitness, mental, cardiometabolic, emotional and cognitive development and musculoskeletal health [1,2]. Recommendations suggest that children engage daily in an average of 60 minutes of at least moderate intensity PA [3]. However, a study using accelerometer data from ten countries suggested that only 2% of girls and 9% of boys comply with the aforementioned guidelines [4].

In Latin America, 20–25% of children and adolescents are overweight or obese [5]. Although Chile is considered a high income country [6], with the highest human development index in the sub-continent [7], Chile is the most unequal of the countries in the Organization for Economic Co-operation and Development, when assessed using the Gini coefficient [8]. This inequality is reflected in children’s obesity and PA opportunities. Although in Chile 41% of adolescents aged 14 and 15 year old in low socioeconomic groups are classified as overweight or obese, only 27% amongst those in the higher socioeconomic groups suffer from these conditions [9]. Similarly, it has been shown that the majority of private schools devote three or more hours per week to physical education classes whereas most public schools dedicate fewer than two hours [10].

In recent years, studies have increasingly shown the strong influence of the environment on children’s activity [11–13] and have considered the amount of time spent outdoors as a surrogate estimate of children’s PA due to its capacity to promote unstructured play [14,15]. Faulkner et al. [16] found that children who spent two or more hours per day playing outside accumulated 27% more minutes of moderate-to-vigorous physical activity (MVPA) than children who played outside for fewer than 30 minutes.

Playing has been recognized worldwide as a right of children [17]. Its characteristics such as adventure, freedom, pleasure, creativity, and risk, are essential for healthy learning and growth, as well as necessary for physical, social, emotional, and cognitive development [18]. As Alexander et al. [19] highlight, play is an activity with benefits which include, but are not limited to, an increase in PA. Play’s broader contributions beyond physical health, such as social health and emotional well-being [20,21] are consistent with the World Health Organization’s definition of health as a state of complete social and mental as well as physical well-being [22]. However, a range of factors during the last two decades have diminished children’s participation in play and spontaneous activities [23]. Creating supportive environments is one of Health Promotion’s five health action areas as established in the Ottawa Charter for Health Promotion [24]. Therefore re-establishing the right to play by providing supportive physical and social environments can be referred to as an integral health promotion strategy.

Few studies have evaluated how environmental interventions at a community level affect outdoor play and children’s PA. Farley et al. [25] found that in a neighborhood that provided supervised schoolyards with improved equipment that opened after school hours there were 30% more active children in comparison to the number of active children in areas without these conditions. Similarly, high attendance rates were reported when pop-up parks with age-appropriate activities were implemented in parking lots [26]. Conversely, during programs in which children were encouraged to participate in outdoor activities without corresponding changes to the environment, the participation rates were low [27].

Worldwide, a number of programs have temporarily turned streets into PA appropriate spaces, with 92% of these interventions taking place in Latin America [28]. Since these generally district-wide programs usually cover several kilometres of main streets, children’s participation relies on the company and vigilance of adults. Play Streets initiatives have also implemented traffic restrictions in several Anglophone and European countries [29–32]. In these programs, self-organized residents of local streets apply for closure permits that allow children to safely play near their houses within an enclosed area.

A study conducted in Belgium evaluated the effect of street play on children’s PA. A significant increase in MVPA was found in those children exposed to the intervention, in comparison to children from control neighborhoods [32]. Another study found a three-fold increase in the proportion of children engaging in vigorous PA during Play Streets events in the US [33]. Both studies have shown promissory findings in regards to Play Streets in developed countries, but have focused specifically on interventions during the summer break, and over a short period of time such as a single event [33], or over one to two weekends [32]. Therefore, the feasibility and effectiveness of a Play Streets programme that takes place during the school semester and that involves weekly events over a longer period of time–and could therefore promote long term behavior change and sustainability of higher PA levels—remain to be tested, particularly in developing countries where opportunities associated with the built environment and policy change are more limited [34].

We designed and implemented ‘Juega en tu Barrio’ (JETB), a street play initiative for low-income neighborhoods. It was inspired by Play Streets programs and adapted locally using the experience and resources of the successful Chilean program ‘CicloRecreoVia’ [35], during which many kilometres of streets are closed to cars on Sundays. The aim of this study was to investigate the feasibility and effect of a street play initiative on children’s outdoor play and PA.

## Methods

### Neighborhood selection

**Fig 1:**
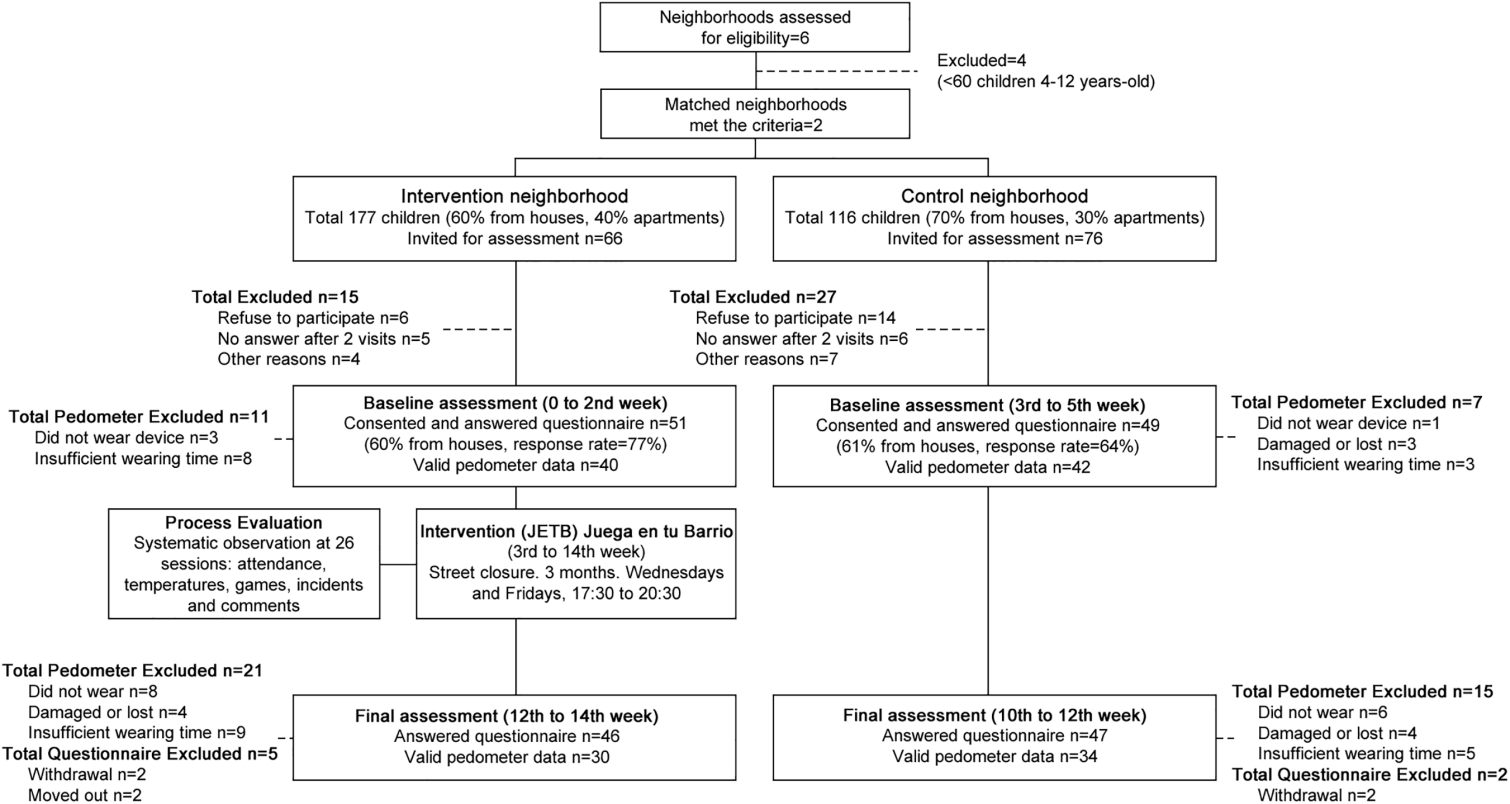
JETB study. Recruitment flow, pedometer use, and questionnaire response rate at each study stage.

Inclusion criteria for both the intervention and control group were: matching socio-economic and environmental characteristics such as proximity to and size ofgreenspace; presence of both apartments and houses, and level of crime. Intervention and control neighborhoods’ data is shown in S1 Table. The presence of at least 80 children between the ages of four and 12, the absence of sport centre alternatives, and separation from each other of at least 1.5 km—to prevent intervention contamination—were also required. For the purpose of this study, a neighborhood was defined as a geographically defined area of four continuous blocks and their immediately adjacent blocks. Six neighborhoods (1191 households) were surveyed to assess neighborhood eligibility according to inclusion criteria. Although we initially planned to randomly select two neighborhoods (as Fig 1 shows) only two out of the six neighborhoods assessed for eligibility met inclusion criteria and therefore became our study groups.

### Setting

JETB was conducted in Santiago. The city has a warm climate and there is a dry season of 7–8 months with rain falling mainly in winter (May to August). The minimum temperature during winter averages 3.9°C and the maximum in summer 29.7°C [36]. The intervention neighborhood was located in a mixed land use-area, with 17,347 inhabitants/km^2^, of which 24% were children. Eighty-five percent of its population were in the two lowest income quintiles [37]. A gated community with six, four-story apartment buildings was located at one end of the neighborhood. Drug dealing activity was common in the street and a shooting occurred nearby before JETB begun. The control neighborhood was located 4.91 km away, in a mixed land use area, with 15,622 inhabitants/km^2^, of which 23% were children. Ninety-three percent of the population were in the two lowest income quintiles [38]. Four, four-story buildings were located at the end of this neighborhood inside a gated community. A meeting with neighbors and stakeholders was held in the intervention neighborhood to obtain input concerning feasibility, acceptability, and design of JETB. The project was well received and the strategies proposed by neighbors such as street cleaning (stones, pieces of glass, street dogs), posting advertising posters, and providing activation aids such as music, facilitators, and playing materials, were included in the intervention. The schedule for the intervention was decided by neighbor consensus.

### Juega en tu Barrio intervention

JETB was implemented for 12 weeks twice per week (September-December 2014). The website www.juegaentubarrio.cl, a logo, and a Facebook page were developed. Four continuous blocks were closed to motorized traffic from 17:30 to 20:30 each Wednesday and Friday. Although the main purpose was to change the neighborhood’s social and physical environment, all levels of the socio-ecological model were targeted.

Individual level: Each child and their family were visited and received an informational leaflet and a magnetic calendar with programmed JETB sessions. The calendar also operated as a self-monitoring instrument to be filled with colored stickers available from organizers at each session. Playing materials (valued at USD$1-$3) such as ropes, kites, paddleballs, diabolos (for juggling), and balls were given to each child.

Interpersonal: Local monitors led group games and incentivized children to meet each other during the first four sessions. Some neighbors took on this role while others provided music and organized contests in some sessions.

Community: The social and physical environment was modified through street closures organized by experienced stewards from CicloRecreoVia, who were located at each intersection. Wearing identifiable uniform and using special traffic signs they rerouted traffic, kept the street free from parked cars, and alerted other adults if any problems arose. Their vigilance also increased the sense of perceived safety, also termed ‘eyes in the street’ [39]. They placed physical barriers at each end of the neighborhood’s main street and cones at every intersection.

Policy: The temporary road closure had local authority permission, which was granted after they met with the research team, and reflects their support towards the intervention and its purpose. The overall intervention cost (resources, uniforms, stewards and coordinator fees) for the 26 sessions was US$2275.

### Study design and assessment

This study included process and outcome evaluations. The former was comprised mainly of neighborhood-level assessments during each session in the intervention neighbourhood in order to study implementation fidelity, attendance, reach, acceptance, barriers, facilitators, and maintenance. A pretest-posttest experimental trial with control group design was used to investigate changes in PA levels and days of outside play. Individual assessments (n = 51 and 49 in the intervention and control neighbourhood respectively) were conducted for this purpose at two timepoints: pre-intervention and during the last two weeks of JETB. The study received approval from the Ethics Committee of the Universidad Metropolitana de Ciencias de la Educación, Chile.

#### Neighborhood-level assessment

Adults and children playing in each block along the closed street were systematically counted during each session by a member of the research team every hour after 18:00. The number of children attending each session was calculated by selecting the daily peak by block amongst the three measurements (18:00, 19:00 and 20:00). To calculate daily peak attendance for the whole street, the peak numbers from each block were added together. The gender of the children was recorded during the last nine sessions. Temperatures provided by the AccuWeather App (AccuWeather Inc, Pennsylvania, USA) were registered each hour and averaged for each day. Each steward registered any comments received from neighbors and the types of games observed at each session.

Eight semi-structured interviews (three before JETB started, five during the intervention and eight after completion) and four focus groups [40] (two during JETB and two after JETB finished, with an average of six participants) were conducted in a non-probabilistic sample chosen to represent different stakeholders from the intervention neighbourhood. The participants were: 30 adults that live in the neighborhood; seven community leaders; six JETB team members, and the district’s municipal social worker assigned to the intervention area. The interviews were transcribed verbatim and analyzed through thematic content analysis, identifying recurrent themes that arose in the data [40]. Topics explored included maintenance after the project ended, and mechanisms that may have favoured or hampered participation (interview guide in S1 File). These findings require in-depth analysis and will form a separate article, however particular topics that could not be quantitatively assessed and that complement the understanding of key aspects of the intervention, are presented in brief.

#### Individual assessment

A responsible adult for each child recruited for the study answered a baseline questionnaire assisted by a trained interviewer which assessed socio-demographic characteristics, parent-perceived social and physical environment (based on questions previously published) [41], and the number of days and duration of weekday outdoor play [42]. In 86% of cases, the respondent was the mother of the child. The responsible adult’s educational attainment was used as a proxy for children’s socio-economic position (SEP). This variable was classified in two dichotomic categories; “high SEP” if the responsible adult completed university or college education and “low SEP” if he/she did not. The same questionnaire was used during the final assessment in both neighborhoods. Questions such as: “What motivated your child to participate in JETB?”, “What activity did JETB replace?” and “Why was JETB useful for your child?” were included in to the final assessment in the intervention neighborhood. Others aimed at appraising changes in parental control, social capital and cohesion such as “Does your child have permission to play in the street without supervision?”, “Did you meet new neighbors during JETB?” and “Do you think the relationships with the neighbors you had already met before JETB became stronger?”. At this stage, children from both groups were also given colour pencils with the instruction to draw “my street” or “I play here”. Anthropometrics were obtained with a portable measuring station (Seca 220, Deutschland, Germany).

PA was assessed by a Movband digital pedometer (Movable, USA). The Movband displayed time as default with step count shown only if a function button was pressed. The Movband has shown high validity when compared with Actigraph accelerometer counts (r = 0.92) and oxygen consumption (r = 0.80) [43]. The device has also shown excellent acceptability in children [44]. The step count function was not explained to the children to prevent pedometer-based PA stimulation. Participants were asked to wear the device for seven consecutive days on the non-dominant wrist during each stage of the study, and were asked to remove it during water-based activities only. The Movbands were charged, synchronized, and downloaded on the same computer to avoid time mismatches. Data were extracted in total steps per hour as presented on the Movable website. A seven-day diary was completed to monitor pedometer wearing time. Data from diaries was merged with pedometer records and filtered for data reduction.

Pedometer-determined PA was considered valid if the participant wore the device for at least 10 hours on three weekdays (including one intervention day) and one weekend day [45]. Steps were summarized as total steps per day as well as total steps during different periods of the day (from 00:00 to 7:59; 8:00 to 12:59; 13:00 to 17:59; 18:00 to 20:59; and 21:00 to 23:59), thus allowing estimation of total steps during JETB intervention. Mean total steps per day were calculated as follows: (mean total steps in weekdays x 5) + (mean total steps in weekend days x 2) / 7. Participants who met the pedometer-derived PA recommendations in children were defined as those participants that recorded at least 13000 steps per day for males, and 12000 steps per day for females [46]. Missing data for pedometer-derived PA were imputed in some children (baseline n = 18; final n = 36) who did not have enough valid days (i.e. forgot to use it in the morning or left it at home) assuming that missing data were missing at random [47]. Missing values in participants were assumed to be similar to those participants with similar age, gender, and body mass index. No differences were observed between those participants that provided valid pedometer data and those who did not in regards to their demographic characteristics.

Participants received a small item to encourage active play after each assessment except at baseline in the control group, where instead gifts were pencil boxes, playdough, paints, and stickers. Written informed consent was obtained from each participant’s responsible adult and children aged seven and upwards.

### Statistical analysis

Mean and range were used to summarize attendance and temperatures. After data were tested for normality, median and quartiles were used to describe the sample characteristics, PA, and playtime outcomes. Wilcoxon’s matched pairs signed-rank test was used to examine baseline–final differences. Differences between groups were examined using Mann-Withney’s U test. Categorical data were shown as percentages and tested using McNemar’s test to examine differences between baseline and endpoint, and Chi Square was used for differences between groups. The level of significance was set at p<0.05. Data cleaning and analyses were completed using SPSS software package, version 16.0.

## Results

In total, 100 children completed the study (51 from intervention neighborhood and 49 from control neighborhood). All participants were Latin, 51% were girls, and 75% were classified as of low SEP. No differences were observed between the intervention neighborhood and control neighborhood for any demographic characteristics except for age, in which participants from the control neighborhood were younger than intervention neighborhood participants. Participant characteristics are displayed in Table 1. Details of recruitment stages, time frames, and response rates are shown in Fig 1.

**Table 1:** Baseline sociodemographic characteristics by trial arm.

	Intervention	Control	
	neighborhood *n* = 51	neighborhood *n* = 49	*p*-value^1^
Age in years; Median (IQR)	9 (5)	7 (5)	0.021
4–8 years; *n* (%)	21 (41.1%)	32 (65%)	
9–12 years *n* (%)	30 (58.8%)	17 (35%)	
Females *n* (%)	24 (47%)	27 (55%)	0.421
Live in apartment *n* (%)	20 (39.2)	19 (18%)	0.964
SEP, low^2^ *n* (%)	39 (76%)	36 (73%)	0.865
BMI; Median (IQR)^3^	19.84 (6.8)	18.2 (5.57)	0.121
Overweight and obese *n* (%)	25 (55.5%)	21 (43.7%)	0.255

*n* = number of children; IQR = Interquartile range; SEP = Socioeconomic position; BMI = Body Mass Index

^1^ *p* values based on Mann Withney U test for continuous data and Chi-square test for categorical data

^2^ Considered low if the responsible adult did not have a college or university degree

^3^Measured in 45 children in intervention neighborhood and 48 in control neighborhood

### Process evaluation

#### Neighborhood level

Twenty four (92%) of the sessions were implemented as planned. Two diverged from the established design. During the second session only two blocks were closed due to a wake, and during the final session a closure event was held instead of regular closure in which neighbors and district authorities gathered to watch artistic performances. Temperatures ranged from 13°C (third session on September 17th) to 33°C (final session on December 5th), with no rain. The most commonly used playing materials were balls (used in all sessions) and ropes used for jumping. The ropes were mainly used in groups and the activity was generally guided by adults (96% of sessions). Average attendance was 60 children (SD = 22) ranging from 29 (Halloween) to 126 (first session, on September 10^th^). Peak attendance tended to be reached toward the later part of the session. Reach (percent of children living in the neighborhood who attended) was 34%. Girls participated more than boys (58% vs. 42% boys). Attendance decreased throughout JETB more noticeably in the block with apartment buildings than in blocks that only had houses. Details of attendance by block and time, and by temperatures are summarized in Table 2.

**Table 2:** Attendance by block, time and temperature, before and throughout the 25 sessions.

	Mean number of children			Mean number of children whole street			Mean number of adults	
Session	Peak^1^ Whole^2^ street (range^3^)	Peak block 1^4^	2+3+4^5^ Block Peak	18:00	19:00	20:00	Peak whole street	Mean temp. (°C)
Before^6^	0	0	0	0	0	0	3	12
1 to 8	78 (35–126)	39	39	47	69	57	43	17
9 to 16	55 (29–68)	21	35	34	44	45	22	22
17 to 25	48 (34–65)	17	31	23	37	43	17	26
**1 to 25**	**60 (29–126)**	**25**	**35**	**34**	**48**	**47**	**26**	**22**

^1^ Children and adults were separately counted in each block at 18:00, 19:00 and 20:00, with the peak calculated as the maximum of the three values

^2^ ‘Whole street’ was considered as the four continuous closed blocks

^3^Minimum and maximum number of children at any session in a month

^4^ First block of the closed street, with houses and apartments

^5^Second, third and fourth (last) blocks of the closed street, all without apartments, only houses

^6^Baseline was assessed two weeks before the intervention started.

Throughout the 26 sessions, 16 positive comments or supportive actions from neighbors and 5 complaints (mainly for noise) were registered, while 26 car drivers complained about traffic detours. Interviews with adult residents suggest that children tend to play only on the same block in which they dwell:

“I don’t give my daughter permission to go over there (blocks further away) without us because people there are sketchy. I try to take care of my children, and people over there try to take care of theirs. There is nothing wrong with the intervention, but here, people from different blocks are not close. The only bad thing here is that we are not close”

(Apartment resident of the closed street, father of a child)

“In our block almost all children play outside, the problem is that the play is segregated. If you pay attention, you’ll see that those who are from down there, play down there, and those that are from over there, play over there.”

(Resident of a house in the closed street)

Although neighbors wanted JETB to continue, it was not maintained by the community after the intervention concluded.

“Everybody liked the intervention. They thought it was good, so they ask why it’s not organized again, why it ended so soon. They wanted it to continue for longer.”

(Apartment resident in the closed street, mother of two children,)

#### Individual level

**Fig 2:**
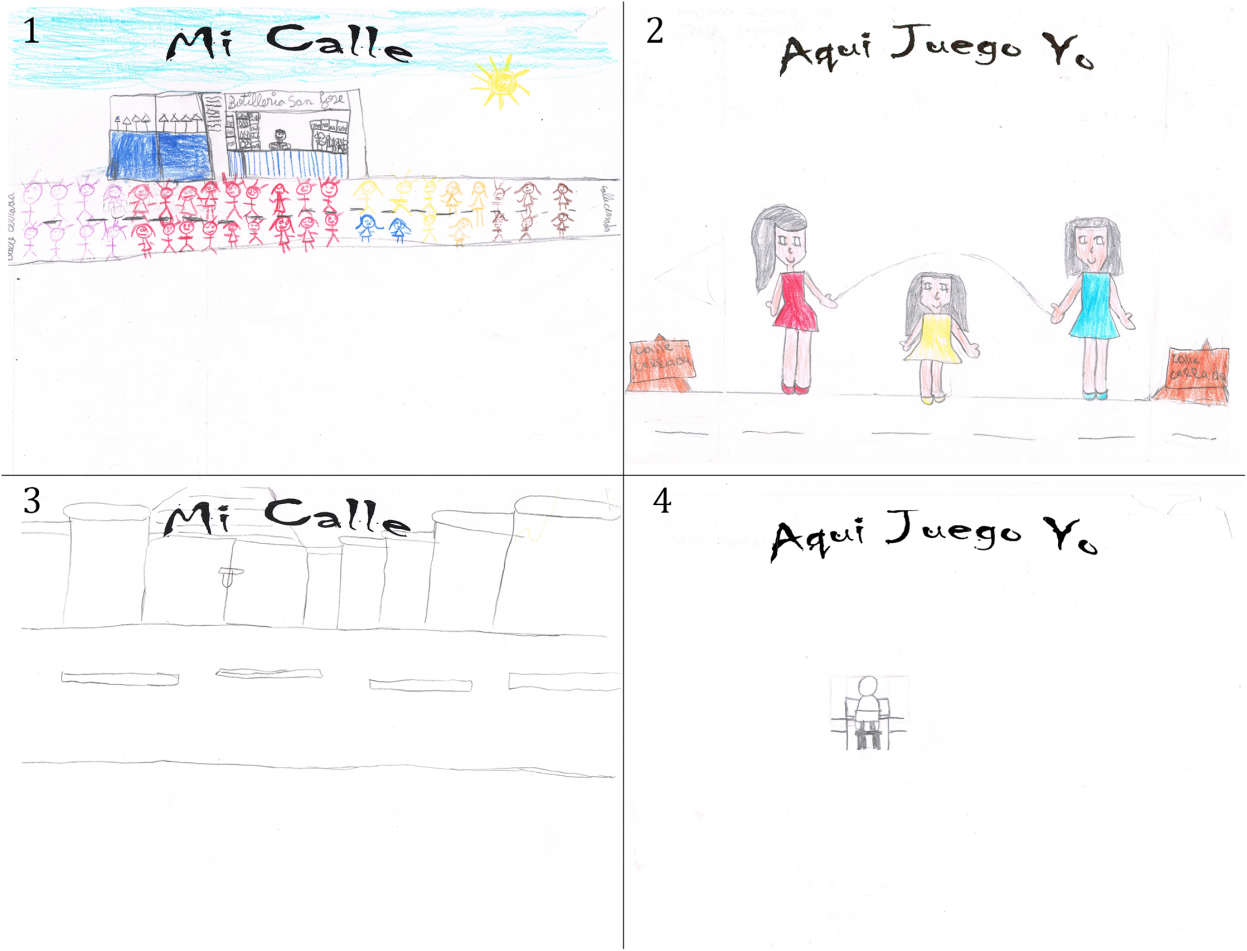
Intervention and control neighbourhood participant’s drawings: Examples of drawings about “Mi Calle” (my street) and “Aquí Juego Yo” (I play here) from the intervention neighborhood (top) and the control neighborhood (bottom) participants. 1 “I drew all of the children playing, my dad’s liquor store, and the house where I live”; 2 “The street is closed, we are skipping with our friends, with Antonia (a steward)”; 3 “It’s the street where I play”; 4 “I drew a little kid playing on the computer. It was me playing on the computer”.

Fifty-three percent of children participated often or always in JETB (more than 70% of the sessions), 31% seldom or never (less than 40% of the sessions), and 16% sometimes (40 to 70% of the sessions). For 59% of children the main motivation for outdoor play was the presence of other children, while JETB replaced screen-based activities for 62% of children. This said, during JETB, screen-based activities remained the number one favorite activity for the children (53% in comparison to 34% who preferred to play with other children when responding about general activity preferences). Before JETB, the main reason parents stated for not letting their children play in the street was traffic/stranger danger (76%). Without the street closure only 4% of children had permission to play in the street without supervision, while 65% had permission when the road was closed to traffic (p<0.001). Thirty-five percent of parents in the intervention neighborhood agreed at baseline that their neighborhood was safe for children to play during daytime. This increased to 54% during JETB (p = 0.07). In the intervention neighborhood, 78% of parents mentioned that they always supervise their children during outdoor play. This proportion did not change with the intervention. In the intervention neighborhood 30% of adults reported meeting new neighbors and 54% strengthened relationships with those they had previously met. When asked how (and if) JETB was useful for children, 36% answered that their child was more sociable/had more friends, while 28% answered that they their child was more independent or self-confident. When analyzing children’s drawings, 35% showed children playing in the street in the intervention neighborhood compared to none in the control neighborhood (Fig 2).

### Physical activity

In the intervention neighborhood significant increases between baseline and final assessments were observed in the number of weekdays with outside play (p = 0.001), after-school outdoor playtime (p = 0.02), and weekly outdoor playtime after-school (p = 0.01). No changes were observed in outdoor play in the control neighborhood, and nor were any differences observed between groups at baseline and final assessments for the same self-reported variables (Table 3).

**Table 3:** Intervention effects on physical activity and outdoor play by study arm.

		Intervention neighborhood			Control neighborhood	
	n	Baseline	Final	N	Baseline	Final
*Outdoor play (parent-reported)*	* *	* *	* *	* *	* *	* *
Number of weekdays with outside play; median (IQR)	46	2 (5)	3 (3)^‡^	47	3 (5)	5 (5)
After-school outdoor playtime^1^ median (IQR)	46	60 (120)	90 (60)^†^	47	60 (120)	60 (90)
Weekly after-school outdoor playtime^2^; median (IQR)	46	120 (480)	300 (480)^†^	47	150 (600)	300 (600)
*Pedometer-derived Physical activity*						
General weekdays^3^; median (IQR)	51	10168 (3797)	12824 (8561)^‡^	49	12107 (5405)	13196 (6071)
Intervention days^4^; median (IQR)	51	13215 (6836)	14124 (12772)	49	12613 (6301)	12165 (7388)
During intervention hours^5^; median (IQR)	32	2090 (2262)	4249 (4942)^‡^	31	2347 (1746)	2911 (2176)
Meeting pedometer-derived physical activity guidelines^6^ n (%)	51	14 (27.5%)	27 (52.9%)^‡^	49	24 (49%)^*^	26 (53%)

† Significantly different from baseline (p<0.05)

‡Significantly different from baseline (p<0.01)

*Significantly different from intervention group

^1^Calculated as min/day

^2^Calculated as min/weekdays

^3^Calculated as steps/day Monday to Sunday

^4^Calculated as steps/day on Wednesday and Friday

^5^Calculated as steps/day on Wednesday and Friday from 18h00 to 21h00

^6^12000 daily steps for girls, 13000 daily steps for boys.

Pedometer-determined PA was significantly different between baseline and final assessment in the intervention neighborhood for daily steps Monday to Sunday (p = 0.006), and steps during the 3-hour intervention (p = 0.004). No significant differences for steps on intervention days were found (p = 0.325). In the control neighborhood no significant differences were found between baseline and final assessments for pedometer-derived PA. A significant increase was observed after the intervention in the percentage of children meeting the pedometer-derived PA recommendations in the intervention neighborhood (p = 0.027) while a small, non-significant increase was observed in the control neighborhood (p = 0.804). Significant differences between groups were observed for the percentage of children meeting the pedometer-derived PA guidelines at baseline (p = 0.027; Table 3).

## Discussion

This intervention was successful in increasing outside play and physical activity in children through an inexpensive and feasible strategy successfully sustained twice a week over a period of three months. It was conducted in a Latin American, mid-low income neighborhood with poor urban infrastructure and high traffic/stranger danger perception regarding street play. The assessment of Play Streets in Belgium [32], as well as other outdoor play studies developed in schools or parks [25,27,48], have been conducted in developed countries. This limits their generalizability to more diverse and disadvantaged settings, which require context-specific interventions in order to enhance PA in children. This study offers relevant information and adds evidence to this field.

Parental safety concerns regarding outdoor play in different contexts have been widely discussed in the literature [49]. In newly industrialized and developing countries, mothers were found to worry about safety more frequently than did those in developed countries [23]. Another study found an association between outdoor play and people being ‘out and about’; if there are more people around, then parents are happier for children to play outdoors [16]. In line with these findings a ratio was constantly observed throughout JETB of around one adult to every three children. Moreover, the proportion of parents who declared that they supervise when their children play out remained equal before and throughout JETB (78%). The increased attendance at the sessions that was observed after 19:00 might also be related to availability of parents to supervise after working hours. These findings suggest that parental concerns were still high in spite of the street closure and the stewards’ presence. Therefore strategies for optimizing neighbor’s trust networks and parents’ vigilance should be taken into account for further interventions in similar settings.

Several factors potentially contributed to increased street play. The number of parents who agreed with the statement “I might give my child permission to play in the street without supervision” rose 61% during the intervention which may show a decrease in perceived traffic danger. Moreover, as the intervention was implemented outside the children’s front doors, it also reduced other common barriers to children’s access to outdoor play in public spaces, such as a lack of independent transportation [50]. However, the number of parents who agreed that their neighborhood was safe for children to play outside in during the daytime increased by only 19% (which was not significant). This may indicate that stranger danger concern was still high. This suggests that higher attendance may be obtained in safer social contexts.

Attendance varied within and across the sessions. It was higher at 20:00 than at 18:00 and this was particularly noticeable during the last month which fell at the end of the year, during summer. This may relate to higher temperatures in the afternoon deterring participation during early hours, as found in a previous study [51]. Additionally, given the increased academic load experienced by children at the end of the semester (the period that coincided with the last month of JETB), parent’s permission for them to play out could be dependent on children having finished their schoolwork, as has been previously suggested [50]. Although attendance during JETB was relatively high compared to other outdoor play interventions [25–27], the decrease in attendance over the sessions during earlier hours may also suggest a wearing-off effect (loss of intervention effectiveness in the medium term), as found by Beulac et al. (2011) [52]. Therefore schedule changes or intermittency could be required if implementation is intended for long periods. Finally, in contrast to previous findings [23,53], attendance amongst girls was higher than that amongst boys. This finding suggests that JETB might be a promising intervention in terms of addressing previously reported gender inequalities in access to PA opportunities [23,54].

Friendship was crucial for the development of JETB as the intervention contributed opportunities for children to be sociable. In line with previous qualitative evidence [55], the presence of other children in the street was quoted as the main driver for children’s participation. This supports previous suggestions to design intervention strategies that enhance sociability [56]. When parents were asked if, and why, JETB was useful, the majority answered that children weremore sociable or had more friends. Thissaid, during JETB, children’s favorite activities were still screen-based activities. This does not match findings from older studies [23,57] in which outdoor play was children’s most enjoyable activity. Given the growing access and time spent in technology-related activities, the benefits provided by Play Streets to children’s emotional and social well-being should be further studied and, as Burdette et al. [58] highlight, broad benefits of outdoor play should be emphasized beyond physical health-related outcomes. Accounting for the range of outdoor play benefits would allow for an accurate cost-effectiveness appraisal of this strategy.

Finding the appropriate size of space for Play Streets interventions may require the balancing of a number of factors. Some studies have shown that the amount of space itself may not be the main factor that increases PA, but rather the programmatic structure within this space is what matters [59]. If only a few blocks are closed, the organization of activities becomes simpler, the project incurs a lower cost, and the number of complaints from drivers may be reduced. On the other hand, during JETB, children tended to play only right in front of their home which is consistent with previous findings [60]. Thus, the larger the closure is, the greater the number of children who might benefit from it. Parental safety concerns that determine if and where children can play in the street must be balanced against the community’s capacity to properly manage any selected length of closure.

### Pedometer-derived PA

A significant increase in total daily steps was found in the intervention neighborhood only, however, no significant difference was found between groups. This could be explained by higher levels of pedometer-derived PA in the control neighborhood at baseline. As PA decreases with age [61], increased PA levels in the control neighborhood could be attributed to age differences, as the average child in the control neighborhood was significantly younger than those included in the intervention neighborhood. The small sample size of this study may also have limited the power to detect significant differences between groups.

Total steps during intervention hours also increased significantly in the intervention neighborhood only. However, mean steps accumulated throughout intervention days did not change significantly. This could be explained by the ‘activitystat’ hypothesis, which proposes that a compensatory change in one domain of PA will occur as a response to changes in another PA domain, therefore maintaining an overall constant level of PA [62]. Thus, children playing in JETB may compensate for increased outdoor PA by arriving home tired and going straight to bed, replacing late evening activities with sleep. These findings are in contrast to D`Haese et al. (2015) whose study showed no compensatory effect during the rest of the day for the increased PA during Play Streets time [32]. Reasons for this difference may be that Play Streets in Belgium were conducted during summer vacations, and finished one hour before JETB sessions, which could favor higher PA levels out of intervention hours. Both studies showed Play Streets contributing a high proportion of daily PA. While JETB—with a duration of three hours—contributed 26% of daily steps (mean 4532, median 4249) the Play Streets study by D’Haese et al.—duration five hours—contributed 53.4% of daily MVPA [32]. Taking into account that the final assessments in JETB were conducted 10 to 12 weeks after the beginning, our results seem valuable as they account for a worn-off effect. This effect was not experienced by the participants of the study of D’Haese et al., in which the intervention lasted only one to two weeks, and in which 50% of participants were assessed as soon as Play Streets started. The number of steps contributed during JETB is also in contrast to after school programs; the latter, with a higher staff to student ratio (1:11), contributed only 2944±1606 steps per day according to Beets et al. [63]. This difference lends support to the consideration of Play Streets as a promising alternative to traditional after-school programs. The proportion of children that achieved pedometer-derived PA guidelines was significantly higher after the intervention in the intervention neighborhood. The increase of 13% in children meeting the PA guidelines which was observed in the intervention neighborhood is relevant when considering the possibility for scaling that this intervention may offer, not only in developing, but also developed countries. Another study implemented after school hours showed similar improvements in children meeting PA guidelines to those observed in our study [64].

### Strengths, limitations, and future research

A novel feature of the intervention was the inclusion of participants living in apartments, which characterizes the urban development of disadvantaged areas most commonly equipped with small or unsafe outdoor spaces. As JETB was conducted amongst an underserved population, our results might be particularly replicable amongst this type of population. The selected setting also prevented the overestimation of effects by imposing unrealistically controlled conditions. Previous Play Streets research had focused on interventions conducted during school vacations [32,33], therefore JETB broadens the evidence available to include interventions run during the school year, providing further data to inform public health practice and policy.

Budgetary limitations restricted the assessment of PA in the subsample to measurement with pedometers instead of accelerometers. This precluded investigating the intensity of the activities performed in JETB and reduced the comparability of the findings. Step count, however, was accounted for each hour, which allowed the estimation of the intervention’s relative contribution to the entire day PA.

Use of other instruments, such as the System for Observing Play and Recreation in Communities (SOPARC) [65] would enhance neighborhood level assessment, accounting not only for attendance, but also type of activity, PA levels, and participants’ age. This, in addition to a larger sample, would allow differential impacts of Play Streets to be studied across diverse age groups. Further research would greatly benefit from the use of accelerometers combined with GPS and GIS, which would account for children’s location, enhancing the accuracy of the estimation of the intervention’s contribution. The relatively high loss rates of devices (11%) experienced in our setting (compared to institutional setting) [66] should be accounted for, when balancing decisions related to instrument selection.

## Conclusion

JETB was a feasible and inexpensive neighborhood-based intervention, pertinent for disadvantaged areas, and capable of fostering outdoor play. It appears to be a promising tool by which to increase children’s achievement of PA recommendations. JETB also appears to be a suitable program capable of tackling disparities in children’s access to activity-friendly environments.

The intervention at children’s front doors allowed for parental supervision, increased permission to play in the street, and stronger social connections amongst children and adults. Thus, the contribution of Play Streets over a wider range of outcomes related to social and emotional well-being should be studied further. Decision-making such as location, size, and schedule should be agreed with the community, accounting for local capacity and need. Further research is required around methods and strategies to promote and support communities to maintain Play Streets.

## Supporting information

S1 Table Comparison of intervention and control neighborhoods’ environmental and social conditions. (DOCX)

S1 File Semi-structured interview guide. (DOCX)
