# Supplementary material for: Reclaiming streets for outdoor play: A process and impact evaluation of “Juega en tu Barrio” (Play in your Neighborhood), an intervention to increase physical activity and opportunities for play
Source: PLoS One. 2017 Jul 3;12(7):e0180172. doi: 10.1371/journal.pone.0180172 (PMC5495338; doi:10.1371/journal.pone.0180172)
Supplement: S1 Table — (DOCX) [file pone.0180172.s001.docx]

**Comparison of intervention and control neighborhoods’ environmental and social conditions**

|  | **Intervention** | **Control** |
| --- | --- | --- |
| Smallest administrative unit, street, and district name | **Unidad Vecinal N13, Calle 13, Peñalolen** | **Unidad Vecinal N24, Calle Los Cerezos, Peñalolen** |
| **Environmental conditions** |  |  |
| Parks^1^ |  |  |
| Quantity | 2 | 2 |
| Park 1: Proximity from selected street | 105m | 5m |
| Total area | 4415m^2^ | 1880m^2^ |
| Amenities and facilities | Playground, benches, grass, and trees | Playground, benches, grass, and trees |
| Park 2: Proximity from selected street | 45 m | 131m |
| Size | 732m^2^ | 1226m^2^ |
| Amenities and facilities | Playground, grass, and trees | Playground, grass, benches, and trees |
| Multipurpose courts^1^ | | |
| Quantity | 2 | 2 |
| Court 1: Proximity from selected street | 90m | 131m |
| Size | 364m^2^ | 441m^2^ |
| Current State^3^ | Poorly maintained | Poorly maintained |
| Availability | Not fenced, free to use. | Fenced, free to use |
| Court 2: Proximity from selected street | 111 m | 131m |
| Size | 516m^2^ | 532m^2^ |
| Current state | Well maintained | Well maintained |
| Availability | Fenced and locked, use reserved for organized activities. Permission required | Fenced and locked, use reserved for organized activities. Permission required |
| Number of schools^1^ | 0 | 0 |
| Land use^2^: Residential | 60% | 91% |
| Mixed | 40% | 9% |
| **Housing** |  |  |
| Good structural condition^2^ | 73% | 78% |
| Type of households^1^ | Mixed (community of houses and gated 4 story apartments) | Mixed (community of houses and gated 4 story apartments) |
| **Socio-demographic conditions^2^** |  |  |
| Crime rate per person | 0.03 | 0.06 |
| Proportion of population in the lowest income quintile | 31% | 19% |

^1^Assesed by JETB research team. The parks and courts described here were those located inside or adjacent to the neighborhoods. The number designated to the parks and courts (1 and 2) were for comparison purposes only.

^2^Obtained from the district´s repository data based on the smallest administrative unit to which each neighborhood belongs [1,2].

^3^ Considered availability of night lighting, and state of fences and ground
