# Supplementary material for: Reclaiming streets for outdoor play: A process and impact evaluation of “Juega en tu Barrio” (Play in your Neighborhood), an intervention to increase physical activity and opportunities for play
Source: PLoS One. 2017 Jul 3;12(7):e0180172. doi: 10.1371/journal.pone.0180172 (PMC5495338; doi:10.1371/journal.pone.0180172)
Supplement: S1 File — (DOCX) [file pone.0180172.s002.docx]

# **Semi-structured Interview Guide**

**A. Pre-intervention interview guide**

1. **General information**

- Name
- Age
- Occupation
- Address
- Number and age of children

1. **Neighborhood**

- How long have you lived in this neighborhood?
- What are your thoughts on the neighborhood? What things do you like/not like about the neighborhood?
  - Follow up questions: safety, security, relationship between neighbors.

1. **Intervention project**

- Do you know about ‘Play in your Neighborhood’?
  - *Explain the intervention to the interviewee if answer is no*.
  - What are your thoughts on this intervention?
- Is there anything you think might strengthen or hinder the intervention?
- Do you think children will get permission to go out and play? Why?
- What good things and bad things do you anticipate happening when ‘Play in your Neighborhood’ starts?
- Do you think the intervention could change how neighbors relate to each other?

**B. During-intervention interview guide**

1. **General information**

- Name
- Age
- Occupation
- Address
- Number and age of children

1. **Neighborhood**

- What are your thoughts on the neighborhood? What things do you like/not like about the neighborhood?
  - Follow up questions: safety, security, relationship between neighbors.
- How would you describe the neighbors’ relationship between one another?
- Is there anything you think could improve the relationships of the people in the neighborhood?

1. **Intervention project**

- What are your thoughts on ‘Play in your neighborhood’? What do you like/not like about it?
- Have you seen any changes in your neighborhood since the project began?
- What has made the intervention difficult?
  - Follow up questions: Consider individual; family; community, environmental factors.
- What is missing from the intervention? / What could improve the intervention?
- What do you think motivates your child (or children in general) to go out and play?
- In your opinion, what has facilitated the children´s participation in the project?
- Why do you think some children do not participate?
- Do you think ‘Play in your Neighborhood’ can be continued by the community after the planned sessions end? What do you think this would depend on?

**C. Post-intervention interview guide**

1. **General information**

- Name
- Age
- Occupation
- Address
- Number and age of children

1. **Neighborhood**

- What are your thoughts on the neighborhood? What things do you like/not like about the neighborhood?
  - Follow up questions: safety, security, relationship between neighbors.
- What are your thoughts on the community’s involvement in the intervention?
- What community characteristics do you think helped/hindered its implementation?

1. **Intervention project**

- What do you think about ‘Play in your Neighborhood’? What did you like/not like about it?
- What is missing from the intervention? / What would improve the intervention?
- Did you hear other people talk about what they didn’t like about the intervention?
- Have you seen any changes in your neighborhood as a result of the intervention?
- Do you think your child (or children in general) benefited from the intervention?
- When children didn’t participate, what do you think was the main reason for this?
- Do you think that this neighborhood has any particular characteristics that could explain the intervention results?
- Do you think this intervention could achieve similar results in other neighborhoods?
- If you were to tell someone else, who does not live in this area, about the intervention, what would you tell them?

1. **Intervention maintenance**

- Do children still go out and play?
- Do you think ‘Play in your Neighborhood’ can be continued by the community after the planned sessions end? What do you think this would depend on?
